# Clinical Abortions

**Published:** 1858-10

**Authors:** 


### EDITORIAL DEPARTMENT.

---

*Criminal Abortions.*—We have been much gratified with the response to the article on this subject which appeared in the September number of this Journal. We conceive that all the feelings of humanity which should animate every man and every Christian, must burn at the contemplation of the extent to which this abuse has been carried. The opinions of the medical press have been sufficiently well indicated by articles which have appeared from time to time in our exchanges, but none of them have suggested any mode of remedy, though they have all joined in indignant and energetic condemnation. Some of the daily papers of this city have noticed our editorial in the last number, agreeing with us in the culpability of such advertisements, but not bringing the matter definitely before the public. It is true indeed, that this is an extremely delicate and disagreeable subject, and one which must be handled with a great deal of care in a secular journal. The necessity is so urgent, however, that we cannot but regard the daily press as unfaithful to the public in not aiding us in our efforts at reformation. There is one exception to this apathy on the part of our daily newspapers; our esteemed correspondent, Dr. N. L. North, called the attention of the editor of the Brooklyn Daily Times to the subject, who has lately devoted to it an able and earnest editorial article, where the subject is handled with great delicacy and judgment, yet bringing the outrage boldly and plainly before the public. Could this example be followed by more of our influential papers, much could be effected which is impossible to us, who appear only before the eyes of the profession. We desire publicly to express our thanks to Dr. North for his kind endorsement of our sentiments, and his coöperation in our labors; with a few more co-workers of his energy and feeling, the task which we know to be so difficult to the conductors of Medical Journals, unaided, though not unapproved in their efforts, by the profession at large, would be comparatively easy. We extract in full the editorial from the Brooklyn Daily Times:

*Criminal Abortions.*—We have been requested by a medical friend, to call attention to an article which appears in the current number of the *Buffalo Medical Journal*, under the above caption. The subject is a delicate one for a family paper to allude to, much less discuss; yet if the allegations of the Journal are true—and our friend assures us that his practice and that of every member of the profession furnishes evidence of their truth—then assuredly no mock delicacy or prudery should be suffered to stand between error and crime, and their needful exposure. No one doubts the propriety of medical journals treating on such a subject; but if the evil is so wide spread as is stated, the medical press, unaided by the newspaper world at large, cannot remove it. It is the family newspaper alone, which reaches the class whose moral sense needs to be awakened on the subject—and therefore it is the general newspaper which must grapple with the evil and strive to overthrow it.

The Journal asserts that the daily, and even the religious press, insert advertisements of professed abortionists; of pills which "are sure to procure abortion," from which "miscarriage will certainly ensue," &c. That in consequence of the continual and unchecked publicity afforded to this class of announcements, females have "come to regard abortion as one of the most innocent and natural things in the world"—so that it is no uncommon thing for medical attendants to be as coolly and unconcernedly asked to produce abortion as a dentist would be requested to draw a tooth. Nay, it is asserted, and advertisements are extracted from popular journals to prove the fact, that instruments are openly announced, and largely used, which are warranted to "regulate or limit offspring, without injuring the constitution." Still further, the Journal does not hesitate to assert that neither the poor, the abandoned, or the ignorant, patronize these infernal inventions so much as the "educated and refined" ladies of fashion and social position.

We cannot but regard the extensive publicity given to advertisements of the class we have referred to, as a conclusive proof that the crime which they facilitate is of frequent commission. No article is largely advertised, for which there is not a large demand—for it is the extent of the demand which warrants the seller in producing his goods and placing them before the public. If, then, abortion is thus practised—extensively and with hardly the thinnest veil of disguise—it would ill become the conductors of the press, who claim to be the guardians of public morals, to conceal the fact, as to squeamishly hesitate in denouncing this class of advertisers as atrocious scoundrels, and any publisher who inserts their advertisements as *particeps criminis.*

Both in law and common sense, infanticide is regarded as murder, and it needs little knowledge of physiology to be aware that abortion is but a form of infanticide. Unless our boasted civilization is to sink to the level of Chinese barbarism, our women, of every class and degree, must be convinced that to deprive their inchoate offspring of life, is to commit murder, and that under circumstances of aggravation which make the foulest of crimes more atrocious.

The "Times" says well, that it is the general newspaper which must grapple and overthrow this evil. This fact is too evident to need discussion. The ridiculous, so-called delicacy which would exclude such a subject from the public gaze, which would whiten this foul and pestilential sepulchre which exists in our midst, its noisesome emanations tainting the moral health of every female, should find no place in the heart of the high-minded guardian of the public morals. While our clergy are denouncing from the pulpit the vice of licentiousness, we have brought to the very heart of every family, and sometimes, as we remarked in our former article, brought there in the columns of papers devoted to religion, flaming announcements of drugs which profess to remove one of the barriers to the indulgence of criminal passions, the fear of *unfortunate consequences.* That this is a safeguard, and at times the only thing which preserves from crime, there can be no doubt; and undesirable as it is that virtue should be preserved by fear of consequences alone, and not by the high moral sentiment which we have been accustomed boastingly to attribute to the American female in a greater degree than to the female of any other nation, we cannot but wish it to be preserved, and pray that it may save those who have ceased to heed the promptings of a more elevated sentiment.

It is useless for us to dilate at greater length upon the effect, on the moral sense of a community, produced by the impression that nothing is more easy than to procure an abortion by the use of internal remedies, and that, too, without danger of life and health; it is our duty to set this matter right in as far as is possible. Every physician knows the hazard of such drugs; and every one has met with cases in his practice which exemplified this fact. It is in this country, however, that such abuses are carried to their greatest extent, and it is not at all improbable that that is one of the causes of the difference in aspect which is frequently noticed between the ladies of our country and of Europe, especially the English ladies. The diseases of the uterus to which the use of "abortion medicine, is so apt to give rise, are much more common here than abroad, and give to the sufferer that peculiar languid aspect and appearance of premature age of which the American women have been accused." We extract the following communication in support of this view from our friend Dr. North to the editor of the *Brooklyn Daily Times:*

Mrs. Harriet Beecher Stowe, in speaking of American and English ladies, says, "How is it that our married ladies dwindle, fade and grow thin—that their noses incline to sharpness, and their elbows to angularity just at the time of life when their Island sisters round out into a comfortable and becoming fullness? If it is the fog and sea coal, why then, I am afraid we never come up with them. But perhaps there may be other causes why a country which starts some of the most beautiful girls in the world, produces so few beautiful women. Have not our close heated stove rooms, something to do with it? Have not—"

But I need not quote further. Mrs. Stowe proceeds to speculate as to the probable causes of this difference, which, evidently are, to a great extent, errors in regimen, diet, &c., &c., &c. Nevertheless allow me to suggest, Mr. Editor, that the subject which you so carefully, yet fearlessly and plainly brought before your readers, in a leader of Saturday last, to wit—Criminal Abortions—may have something to do in producing this alarming degeneracy of our American married women. Permit me to say, that as a medical man, I *know* this to be a fact, and more, that while the physical frame is undergoing premature decay, the natural delicacy, the moral purity and moral integrity are fast ebbing out, to be replaced by a *false* delicacy, an *assumed* modesty, a *prudery* in fact, which is intended to cover up the whole subject, and the legion of Demons—male and female—who advertise their "never failing" pills and instruments are careful to *encourage* this depraved delicacy, and first and foremost to join in a shriek of holy horror at the idea of mentioning the matter under *any* circumstances whatever, and at the utter impropriety and inconsistency of discussing the subject in a sterling family journal.

MEDICUS.

We have almost made another long article upon the subject, which we fear will fatigue some of our readers. If our enthusiasm has led us to extremes, we must be forgiven an account of the good cause in which we are engaged. Some of our professional brethren may not join with us in our opinion of the criminality in killing a fœtus. In our first article, we said that there was really no difference between the crime of taking the life of a fœtus at three months, a newly born infant, or even an adult human being. The first offence seems to us as henious as the last; but there is an unaturalness about the production of abortion, which inspires a disgust which we feel for no other crime. In a purely philosophical point of view, it is impossible to say that the destruction of the life of an ovum even the very hour after impregnation, is less a sin than to destroy it at the second or third month, for, in the natural course of things, the one is as capable of becoming a being endowed with an intellect and a soul, as the other; and yet, even *we* cannot but see a difference here, if not in a physiological, at least in a moral sense, and we could never, at all events, be able to convince the public that there was any great amount of crime in destroying the vitality of a newly impregnated ovum. As it is not alone to the physician that we appeal, we will not discuss this point. But true it is, that when the mother begins to feel that she has a living being within her, that is of her own flesh and blood; when instead of the feelings of fond anticipation which we should expect, and which is so natural, we have the time looked forward to with horror, when the *pleasures of a gay life* will be interrupted by maternal duties; then, when the child is thought of as a human being, when its development and vitality cause sorrow instead of joy, we should need no argument to convince the learned or the unlearned that the takirg of life is a crime.

In accordance with the plan which was suggested in the Sept. number, for the suppression of abortion advertisements, Prof. White cffered the following resolutions at the regular meeting of the *Buffalo Medical Association* with a wish that their consideration should be deferred until the next meeting:

"*Resolved*, That the Association fully concurs in the sentiments expressed in the editorial article, entitled "Criminal Abortions," in the Sept. number of the Buffalo Medical Jonrnal, relative to its frequency and criminality.

"*Resolved*, That a committee of three be appointed from this Association to confer with the county and city authorities as to the laws now existing, if properly enforced, and whether further legislation is necessary for the abolishment of this great and growing evil.

"*Resolved*, That this committe invite the coöperation of the Medical Societies and Associations in this State, in any measure which may be deemed necessary and expedient to lessen these horrible offences against the morality of the community."

This is precisely what we desired, and nothing can be more certain, than if every medical society should have similar resolutions brought before it, and act with unanimity on this subject, that great good would be effected. We here entreat the coöperation of medical societies in this State, and are sure that their example will be followed by others. Let not the profession look on this subject with apathy, but one and all make a strong effort to root out this evil, and they *will* root it out. We do not advise a Quixotic attempt to have abortionists hung, drawn and quartered, but merely wish to begin the matter by keeping their vile announcements out of respectable daily papers; and if this cannot be done we are powerless indeed; if it can be done, and we have the most enduring faith in its possibility, are we men, are we Christians, are we good physicians, if we fail to make the effort?

